# Effectiveness in Bowel Cleansing and Patient Tolerability of Polyethylene Glycol versus Sodium Picosulphate in Patients Undergoing Colonoscopy

**DOI:** 10.1155/2020/1234341

**Published:** 2020-05-30

**Authors:** Amit Kumar Jaiswal, Shatdal Chaudhary

**Affiliations:** ^1^Department of Internal Medicine, Siddhartha Nagar City Hospital, Bhairahawa, Nepal; ^2^Department of Internal Medicine, Universal College of Medical Sciences, Bhairahawa, Nepal

## Abstract

**Introduction:**

Colonoscopy is considered as a gold standard investigation for screening of colorectal cancer and other lower gastrointestinal pathologies. Adequate bowel preparation is absolutely necessary for a fruitful colonoscopy. Various bowel cleansing agents are being used for his purpose. The aim of the present study was to compare the two bowel cleansing agents: a single dose of Polyethylene Glycol (PEG) solution and a split dose of Sodium Picosulfate (Na PICOSUL) tablet with regards to cleansing efficacy and tolerability among the patients scheduled for colonoscopy.

**Methods:**

It is an open-label hospital-based observational study. A total of sixty-four patients were grouped randomly into two groups of bowel cleansing agents that are PEG and Na PICOSUL during the study period between 1^st^ December 2015 and 30^th^ November 2016. Patients' tolerability was evaluated using a structured questionnaire, and the bowel cleansing efficacy was evaluated using the Aronchick Bowel Preparation Scale (ABPS).

**Results:**

The group that received PEG solution was found to have better efficacy than that which received Na PICOSUL tablet (63.3% versus 29.4%, respectively, with a *P* value < 0.028) with excellent grade as per ABPS. The Na PICOSUL group was found better in terms of tolerability than the PEG group as nausea/vomiting was encountered significantly higher in the PEG group than in the Na PICOSUL group (43.3% versus 11.8%, respectively, with a *P* value < 0.01).

**Conclusions:**

Colonic preparation with a split dose of Na PICOSUL tablet was better tolerated than the evening before regimen of PEG solution. However, PEG solution was found to be more efficacious in bowel cleansing, but procedural performance and lesion detection were similar for both agents.

## 1. Introduction

Colorectal cancer (CRC) is a commonly encountered malignancy worldwide and accounts for over 9% of all cancer incidences [1]. It is also one of the common cancers in Nepal [2]. Colonoscopy is widely used for the diagnosis and treatment of colonic disorders. Properly performed colonoscopy is generally safe and well-tolerated by most patients. It visualizes the mucosa of the entire large intestine and distal terminal ileum. Colonoscopy is the preferred method to evaluate the colon in most adult patients with lower gastrointestinal symptoms, iron deficiency anaemia, abnormal radiographic studies of the colon, positive colorectal cancer screening tests, post-polypectomy and post-cancer resection surveillance, surveillance in inflammatory bowel disease, and in those with suspected masses [3]. Recent technological advances in colonoscopy have led to improvements in both image enhancement and procedural performance. However, the utility of these technological advancements remains dependent on the quality of bowel preparation. Poor bowel preparation has been shown to be associated with reduced caecal intubation rates, increased patient discomfort, and lower adenoma detection [4].

Polyethylene glycol (PEG) is one of the commonest methods of cleansing the colon, but it needs to be taken in large volume which is cumbersome [5]. Sodium picosulfate (Na PICOSUL) can also be used as an alternative for bowel preparation where it is taken orally along with large volume of clear fluids or oral rehydration solution [6]. This study was conducted to compare routinely used single-dose high-volume PEG solution with split dosing of Na PICOSUL tablets as an alternative in terms of patient tolerance and bowel cleansing efficacy in patients undergoing colonoscopy.

## 2. Methods

It was a single-centre hospital-based open label observational study carried out at the endoscopy unit of Universal College of Medical Sciences Teaching Hospital (UCMS-TH), Bhairahawa, Rupandehi, Nepal. The study was carried out from 1^st^ December 2015 to 30^th^ November 2016. The study was cleared by the ethical review committee of the institute. Written informed consent was taken from all the patients. There were total 64 patients included in this study.

### 2.1. Inclusion Criteria

- All patients aged >16 years who were subjected to colonoscopy in endoscopy unit of UCMS-TH during the study period
- The patients who provided written informed consent for the procedure

### 2.2. Exclusion Criteria

- Patients who refused to provide written informed consent for the study
- Patients aged ≤16 years
- Patients unfit for colonoscopy or who are hemodynamically unstable (e.g., severe shock, recent myocardial infarction, unstable angina, cardiac arrhythmia, severe respiratory disease, possible visceral perforation, uncooperative patients, and unconscious patients)

### 2.3. Grouping of the Population under Study

All the participants (*n* = 64) were categorized into two groups based on given agent types (group 1: polyethylene glycol solution (PEG) and group 2: sodium picosulfate tablets (Na PICOSUL)). A simple randomization with the coin flip method was used to allot each individual into the abovementioned two groups where heads stood for PEG and tails for Na PICOSUL.

The single-dose regimen of PEG solution for colonoscopy was considered as the control group for the study, while the patients in Na PICOSUL were considered as the intervention group. Both groups were compared in terms of efficacy and tolerability measuring the frequency of the respondents for each parameters and sub-parameters. Patients were inquired in details about their indication for colonoscopy, duration of their symptoms, co-morbid condition, and smoking history and also vital statistics like height and weight were measured. After randomization to their respective groups, patients were given a nearest suitable appointment for colonoscopy. Patients were explained in details about the instruction for use and the method of consumption of the bowel cleansing agent they were randomized to by the endoscopy unit nurse. Patients were advised to avoid iron preparation and seeds during the week before colonoscopy, if the procedure was elective. If the patients were taking insulin or oral hypoglycemic agents (OHA), they were asked to abort the dose the evening before the procedure and in the morning on the day of the procedure. Patients were advised to take a low-fiber diet in the morning meal and only clear liquids from the evening the day before the procedure.

### 2.4. Application of Cleansing Agents to the Study Population

Under this study, two types of bowel cleansing agents were used: (i) PEG (PEGLEC® Trade mark of TABLETS India limited under the license of ROUSSEL MORISHITA Company limited, Osaka, Japan) and (ii) Na PICOSUL (Tab. Cremalax® 10 mg manufactured by Acme foundation private limited® Trademark of Abbott group of companies, Solan, India). The patients in group 1 were prescribed to mix one sachet of PEG solution with 2 litres of water and were asked to take it from 6 pm to 9 pm on the day before colonoscopy and were also asked to take additional 2 litres of the electrolyte solution prepared by mixing 2 sachets of Electrobion powder (®Merck Pharmaceuticals) with 2 litres of water to be consumed during the same time period to prevent dyselectrolytemia. In group 2, the patients were prescribed to take two doses of 10 mg NaPICOSUL tablet with two litres of the electrolyte solution to be consumed with each dose. The first dose was prescribed to be taken from 6 pm to 9 pm on the day before colonoscopy, and second dose was prescribed to be taken from 5 am to 7 am on the day of colonoscopy.

All the patients were advised to adhere to the prescribed regimen, and those who did not comply with the prescribed regimen were rescheduled for the procedure to another date. Preprocedural vital signs like blood pressure, pulse rate, and respiratory rate were measured on the day of colonoscopy just prior to the procedure. Patient tolerance to the prescribed regimen was evaluated by using a simple questionnaire which was filled up by the patient himself/herself if he/she is a literate or filled by the endoscopy nurse if a patient was not formally educated. The discomfort experienced such as nausea/vomiting, bloating, incontinence, abdominal pain/cramps, and overall discomfort was rated on a 3-point subjective analogue scale as follows: grade 1 with mild discomfort, grade 2 with moderate discomfort, and grade 3 with severe discomfort.

This was recorded as their mere presence or absence anytime during the study after consumption of the bowel cleansing agents and before the colonoscopy procedure. The colonoscopy procedure was performed using a FUJINON EVE®EPX-201H processor and FUJINON EC-250WL5 endoscope. The colonoscopy procedure was performed single-handed by an experienced endoscopist who has performed a large number of colonoscopies in the last seven years.

The quality of bowel cleansing was assessed using the Aronchick Bowel Preparation Scale (ABPS), [7] a validated bowel preparation score, to rate the quality of bowel preparation. A small volume of clear fluid or >95% of the surface seen was considered as “excellent,” clear liquid covering 5–25% of the surface and some semisolid stool suctioned or washed away during colonoscopy procedure but >90% of the surface seen were defined as “good” and “fair,” and semisolid stool that could not be suctioned or washed away with <90% of the surface seen was defined as “poor” [7]. Any complication during the procedure such as bowel perforation, aspiration, or any adverse event during and within 48 hours (through telephone) after the procedure was registered. All the data were collected and filled in the proforma.The compiled data from the proforma was entered in the computer in the windows office version 10 excel spread sheet and transferred to SPSS version 20. Statistical analysis was conducted using SPSS for windows software. Categorical data were expressed as the corresponding percentage. The Pearson chi-square test was used for testing relationships between the categorical variables. The level of significance for all analytical tests was set at 0.05 where a *P* value < 0.05 was considered to be statistically significant.

## 3. Results

The total of sixty-four (*n* = 64) patients were randomized, of which thirty (*n* = 30) were randomly allotted to the PEG group (Group 1) and thirty-four (*n* = 34) to the NaPICOSUL group (Group 2). The mean age was 45.58 ± 16.71 years, and the median was 46 years. The age of the population patients ranged from 17 years to 81 years. Among them, twenty-six (*n* = 26) were of ≥50 years where 13 (43.3%) belonged to group 1 and 13 (38.2%) belonged to group 2. Similarly, in the age group of 40–49 years, a total of eleven (*n* = 11) patients were included, out of which 4 (13.3%) belonged to group 1 and 7 (20.6%) belonged to group 2. Likewise, of the fifteen (*n* = 15) patients belonging to the age group of 30–39 years, 9 (30%) were allotted to group 1 whereas 6 (17.6%) were allotted to group 2. From the nine patients (*n* = 9) of age group of 20–29 years, 3 (10%) were placed in group 1 and 6 (17.6%) in group 2. The least population (*n* = 3) in the study were of <20 years, where 1 (3.3%) was allotted to group 1 and 2 (5.9%) in group 2. There were 38 (59%) males and 26 (41%) females. Out of the 30 patients in group 1, there were 16 males (53.3%) and 14 females (46.7%). Similarly, out of 34 patients in group 2, there were 19 males (55.88%) and 15 females (44.22%). Patients from the study population presented to us were with various symptoms. The most common indication for colonoscopy in both groups was chronic diarrhoea (36.66% and 35.29% in group 1 and 2, respectively) followed by constipation (26.66% and 26.47%), visible blood (23.35% and 23.54%), and abdominal pain (13.33% and 14.70%) (Table 1).

### 3.1. Patient Tolerability

**Figure 1:**
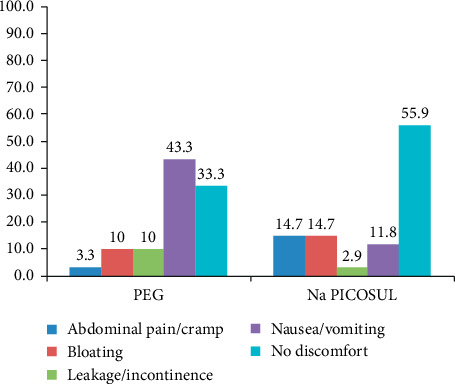
Tolerability of the bowel preparation.

Though there were three levels of discomfort included in the study as grade 1 with mild discomfort, grade 2 with moderate discomfort, and grade 3 with severe discomfort, there was no response received for the moderate and severe levels of discomfort. Therefore, the comparison of outcome has been made only for mild discomfort. The outcome of the bowel preparation for the two groups is presented in Figure 1.

Among the five parameters used to rate tolerability, the significant difference between the two types of agent was found only for nausea and vomiting (Table 2) where 43.3% of the patients in the PEG group (group (1) were reported to have nausea and vomiting whereas only 11.8% of the respondents expressed such problem due to the use of Na PICOSUL (group (2) which was statistically significant (*P* value < 0.004).

Of the total 64 patients, i.e., 30 in group 1 and 34 in group 2 were prescribed to consume a total of 4 L of the electrolyte solution along with the allotted bowel cleansing agent. Only two patients in the group 2 did not adhere to the prescribed protocol and so, were rescheduled for the procedure to another suitable date.

### 3.2. Bowel Cleansing Efficacy

The quality of bowel cleansing as assessed by the Endoscopist revealed that PEG group patients had better cleansing quality as compared to the Na PICOSUL group patients (63.3% vs. 29.4%) with “excellent” grading on ABPS (*P* value = 0.028) which was statistically significant (Table 3).

### 3.3. Colonoscopy Performance

The procedure was complete in 59 (92.18%) patients, i.e., 28 (93.33%) and 31 (91.17%) in group 1 and group 2, respectively, and the difference was not statistically significant (*P* value = 1). During the procedure, in 2 (6.66%) in group 1 and 3 (8.82%) in group 2, the colonoscope could not be passed beyond the splenic flexure due to inability to visualize the colonic mucosa and were rated as “Inadequate” (Aronchick score 5).

Significant lesions (anal canal or rectum or sigmoid colon) were found in 31 patients (48.43%) out of 64. There were 5 cases of ulcerative colitis which were confirmed by histopathology. Among them, in three patients, disease was involving the rectum and sigmoid, and in two patients, there was pancolitis. The most common finding was nonspecific colitis and hemorrhoid. Hemorrhoids were seen in 4 (13.35%) patients in group 1 and 6 (17.64%) in group 2. Similarly, non-specific colitis was seen in 5 (16.66%) patients in the PEG group and 5 (14.70%) patients in the Na PICOSUL group (Table 4).

No major colonoscopy-related complication was noted which requires any specific treatment during and 48 hours after the procedure.

## 4. Discussion

Out of sixty-four patients included in the study, there were thirty-eight males (59%) and twenty-six females (41%). The male and female ratio was about 1.5 : 1. The mean age of the patients was 46 ± 16.71 years. Results are similar to those of another study where most of the patients were male [8]. Similar results were also found in the study conducted by Leitao et al. [9].

The most common indication for colonoscopy in the present study was chronic diarrhoea followed by constipation, bloody diarrhoea, and abdominal pain. A study conducted by Chaudhary et al. has demonstrated that altered bowel habit, chronic diarrhoea, and bloody diarrhoea were the three most common indications for colonoscopy [10]. Another study by Kim et al. has also found that colorectal disease was the most common indication for colonoscopy [11]. Similarly, a study conducted by Leitao et al. has found that visible blood was the most common indication for colonoscopy [9]. In another study, it was found that the most frequent reason for colonoscopy was rectorrhagia (53.6%) followed of the abdominal pain (30.4%) [12].

Patient tolerance to bowel cleansing was assessed by measures of outcome experienced by patients themselves using a simple questionnaire completed just before the colonoscopy. Nausea and vomiting were significantly more in the PEG group (43.3%) than in the Na PICOSUL group (13.8%) which was found statistically significant (*P* value < 0.004). Patients in the Na PICOSUL group were reported to have more incidence of abdominal pain/cramp 5 (14.7%) vs. 1 (3.3%) in the PEG group, but the difference was not statistically significant (*P* value = 0.259). A study conducted by Kojecky et al. shows that a PMC-based solution was generally better tolerated than PEG regardless of the regimen used (*P* value < 0.001). Nausea was reported mostly after the 4 L PEG (32.8%, *P* value < 0.001), incontinence after a split PMC dose (34.4%, *P* value = 0.002), and bloating after the 4 L PEG (38.0%, *P* value < 0.001) [13]. Another study conducted by Leitao et al. has found that nausea and vomiting were significantly more frequent among participants in the PEG group (*P* value = 0.001), and the overall experience was significantly worse (*P* value = 0.007) compared to PMC [9]. Similarly, a study conducted by Manes G. et al. has found that PMC was better tolerated than PEG + Ascorbic acid (PEG + ASC), as evidenced by the significantly higher number of patients who described no or mild discomfort from the preparation (136/140 (97.1%) vs. 123/145 (84.8%); *P* value < 0.0003) Patients who received PMC had significantly less bloating, belching, nausea, and vomiting, but significantly more hunger [8]. It is seen that PEG is not well-tolerated among the patients undergoing colonoscopy due to the palatability and large volume of fluid with which it is meant to be taken compared to other bowel cleansing agents.

The main finding in our study concluded that PEG was superior to Na PICOSUL in terms of efficacy as measured by ABPS. The quality of bowel cleansing as assessed by the endoscopist revealed that the PEG group patients had better cleansing quality as compared to the Na PICOSUL group patients with a *P* value 0.007 which was statistically significant, and 63.3% vs. 29.4% were graded as “excellent” Aronchick score 1 (*P* value = 0.028) which was also statistically significant. In another study, satisfactory bowel cleansing (Aronchick score 1 and 2) was significantly more frequent when a split dose was used irrespective of the solution type (*P* = 0.024). In single dose regimens, PMC performed better than PEG (82.6% vs. 73.0%). Single- or split-dose PMC preparations were comparable [13].

In a study conducted by Hassan et al., among five meta-analyses of head-to-head comparisons of PEG vs. oral Na PICOSUL, three concluded that satisfactory (excellent or good) colon cleansing is significantly less frequent with PEG as compared with oral Na PICOSUL (70%–77% vs. 75%–82%). The two remaining meta-analyses found no statistically significant difference between PEG and oral Na PICOSUL for overall colon cleansing [14]. A study conducted by Leitao et al. has found no significant difference between the two groups with regard to the overall cleansing effect [9]. Similarly, a study conducted by Manes et al. has found that the split regimen was more effective in terms of bowel cleansing compared to the same day regimen [8].

The procedure was complete in 59 (92.18%) patients, 28 (93.33%) and 31 (91.17%) in the PEG group and Na PICOSUL group, respectively, and *P* value = 1 which was defined by the ability to visualize caecum or terminal ileum. In 3 patients, 1 (3.3%) in the PEG group and 2 (5.9%) in the Na PICOSUL group, the colonoscope could not be passed beyond the splenic flexure due to inability to visualize the colonic mucosa and were rated as “Inadequate” Aronchick score 5. The difference was not statistically significant. A study conducted by Leitao et al. has also showed similar results [9].

Colonoscopy performance is rated by the ability of the colonoscope to visualize the caecum or terminal ileum which in part is mostly governed by the bowel cleansing efficacy. So, the choice of the bowel cleansing agent becomes pivotal to rate the colonoscopic procedural performance. Other factors such as position of patient, level of sedation, age, and comorbid status of the patient also play a role in procedural performance. Hence, the choice of bowel preparation should be individualized according to patient comorbidity status and efficacy of the bowel cleansing agent used.

This study is an effort to make a choice of a bowel cleansing agent in terms of the dosing regimen, patient tolerability, and efficacy. However, the choice of the bowel cleansing agent should be individualized keeping in mind the patient tolerability, side-effect profile, patient age, and comorbid status. This study is the first of its kind in Nepal to the best of our knowledge which has compared the two commonly used bowel cleansing agents in terms of patients' tolerability and efficacy. The results of this study will help us to select a proper bowel cleansing agent before colonoscopy. There are certain limitations in this study. As this is a hospital-based single-centre study, the sample size was relatively small. Due to the lack of fund, baseline and postprocedural renal gunction test, liver function test, and electrolyte levels test could not be performed.

## 5. Conclusions

PEG solution is found to be better than Na PICOSUL in terms of efficacy as graded by using ABPS, whereas Na PICOSUL has better tolerability profile. However, colonoscopic performance and lesion detection were found to be similar in both groups. PEG is found more efficacious than Na PICOSUL in terms of overall bowel cleansing. However, both PEG and Na PICOSUL were found quite effective for lesion detection. Therefore, PEG should be the first choice as a bowel cleansing agent for colonoscopy. Nonetheless, Na PICOSUL could be considered as a good alternative in terms of better tolerability.

## Figures and Tables

**Table 1:** Indication for colonoscopy of patients under study.

Indication	Frequency *N* (%)	
	PEG	Na PICOSUL
Abdominal pain	4 (13.33%)	5 (14.70%)
Constipation	8 (26.66%)	9 (26.47%)
Chronic diarrhoea	11 (36.66%)	12 (35.29%)
Visible blood	7 (23.35%)	8 (23.54%)
Total	30 (100%)	34 (100%)

**Table 2:** Patient tolerance to the bowel cleansing agent.

Outcome	Cleansing agent		Total	*P* value
	PEG	Na PICOSUL		
Abdominal pain/cramp	1 (3.3%)	5 (14.7%)	6 (9.4%)	0.259
Bloating	3 (10.0%)	5 (14.7%)	8 (12.5%)	0.85
Leakage/incontinence	3 (10.0%)	1 (2.9%)	4 (6.2%)	0.518
Nausea/vomiting	13 (43.3%)	4 (11.8%)	17 (26.6%)	0.004
No discomfort	10 (33.3%)	19 (55.9%)	29 (45.3%)	0.071
Total cases	30 (100%)	34 (100%)	64 (100%)	

**Table 3:** Bowel cleansing efficacy assessed by using the Aronchick Bowel Preparation Scale.

Aronchick scale	Cleansing agent		Total	*P* value
	PEG	Na PICOSUL		
Excellent (1)	19 (63.3%)	10 (29.4%)	29 (45.3%)	0.028
Good (2)	5 (16.7%)	5 (14.7%)	10 (15.6%)	
Fair (3)	3 (10.0%)	14 (41.2%)	17 (26.6%)	
Poor (4)	2 (6.7%)	3 (8.8%)	5 (7.8%)	
Inadequate (5)	1 (3.3%)	2 (5.9%)	3 (4.7%)	
Total cases	30 (100%)	34 (100%)	64 (100%)	

**Table 4:** Colonoscopy findings in group 1 and group 2.

Colonoscopy findings	PEG	Na PICOSUL	*P* value
Normal	15 (50%)	15 (44.11%)	1
Nonspecific colitis	5 (16.66%)	5 (14.80%)	0.829
Ulcerative colitis	2 (6.66%)	3 (8.82%)	1
Hemorrhoid	4 (13.35%)	6 (17.64%)	0.635
Polyp	3 (10%)	4 (11.76%	1
Carcinoma colon	1 (3.33%)	1 (2.34%)	1

## Data Availability

All the raw data will be made available if needed.
